# Intra-arterial versus standard intravenous administration of lutetium-177-DOTA-octreotate in patients with NET liver metastases: study protocol for a multicenter, randomized controlled trial (LUTIA trial)

**DOI:** 10.1186/s13063-019-3888-0

**Published:** 2020-02-05

**Authors:** Sander C. Ebbers, Arthur J. A. T. Braat, Adriaan Moelker, Marcel P. M. Stokkel, Marnix G. E. H. Lam, Maarten W. Barentsz

**Affiliations:** Department of Radiology and Nuclear Medicine, University Medical Center Utrecht, Heidelberglaan 100, 3584 CX Utrecht, The Netherlands

**Keywords:** Intra-arterial, PRRT, NET, Neuroendocrine tumor, Neuroendocrine neoplasm, Lutetium-177-DOTATATE, ^177^Lu-DOTATATE

## Abstract

**Background:**

Lutetium-177-DOTA-octreotate (^177^Lu-DOTATATE) significantly increases survival and response rates in patients with grade I and grade II neuroendocrine tumors (NETs). However, survival and response rates are significantly lower in patients with bulky liver metastases. Increasing the tumor-absorbed dose in liver metastases may improve response to ^177^Lu-DOTATATE. The LUTIA (Lutetium Intra-Arterial) study aims to increase the tumor-absorbed dose in liver metastases by intra-arterial (IA) administration of ^177^Lu-DOTATATE, compared to conventional intravenous (IV) administration.

**Methods:**

A multicenter, within-patient randomized controlled trial (RCT) in 26 patients with progressive, liver-dominant, unresectable grade I or grade II NET will be conducted. Patients with bilobar bulky disease will be randomly allocated to receive IA treatment into either the left or the right hepatic artery. Using this approach, one liver lobe will be treated intra-arterially (first-pass effect), while the contralateral lobe will receive an intravenous treatment as a second-pass effect. The primary endpoint of this study is the difference in tumor-to-non-tumor ratio of ^177^Lu-DOTATATE uptake between the two liver lobes on post-treatment SPECT/CT (IA versus IV). Secondary endpoints include absorbed dose in both liver lobes, tumor response, dose-response relationship, toxicity, uptake in extrahepatic lesions, and renal uptake.

**Discussion:**

This multicenter, within-patient RCT will investigate whether IA administration of ^177^Lu-DOTATATE results in a higher activity concentration in liver metastases compared to IV administration.

**Trial registration:**

ClinicalTrials.gov, NCT03590119. Registered on 17 July 2018.

## Background

Peptide receptor radionuclide therapy (PRRT) with lutetium-177-DOTA-octreotate (^177^Lu-DOTATATE; Lutathera, Advanced Accelerator Applications, Saint-Genis-Pouilly, France) is the standard treatment for inoperable metastasized grade I and grade II gastrointestinal neuroendocrine tumors (NETs) [1]. Grade I and grade II NETs are well or moderately differentiated tumors. However, metastatic disease at initial diagnosis is present in approximately 50% of patients [2, 3]. The liver is the most frequently affected site of metastases, followed by the peritoneum, bone, lung, and ovary [4]. In order to control disease progression, to achieve symptomatic relief, and to prolong survival, PRRT is increasingly used in the treatment of progressive NETs [5, 6]. ^177^Lu-DOTATATE is a radiopharmaceutical, consisting of a somatostatin-analog peptide labeled with an isotope with a high-energy beta emission, that binds with high affinity to the somatostatin receptor subtype 2 (SSTR-2), overexpressed in grade I and II NET cells [7, 8]. ^177^Lu-DOTATATE significantly increases progression-free survival (PFS) and overall survival (OS), with limited toxicity, compared to non-radioactive high-dose somatostatin analogs in patients with advanced stage NET [5, 6, 9–11].

However, patients with liver metastases have a significantly lower PFS, even after ^177^Lu-DOTATATE treatment [12, 13]. A post hoc analysis of the NETTER-1 trial showed that the presence of bulky lesions (> 3 cm) was significantly associated with a worse PFS after PRRT [14].

To improve the absorption and binding of the radiopharmaceutical to NET cells, small studies have evaluated the effect of intra-arterial (IA) administration of several different radiopharmaceuticals (e.g., ^177^Lu-DOTATATE, ^90^Y-DOTATOC, etc.) into the common hepatic artery [15]. Tumor-absorbed doses seemed to be higher after IA administration of the radiopharmaceutical, but comparative studies have not been performed [15].

The current study compares IA administration of ^177^Lu-DOTATATE with intravenous (IV) administration. This manuscript provides a detailed description of the study protocol.

## Methods

### Hypothesis

The hypothesis is that IA administration of ^177^Lu-DOTATATE into the hepatic artery will result in a higher tumor-to-non-tumor (T/N) uptake ratio, compared to IV administration.

### Trial design

**Fig. 1:**
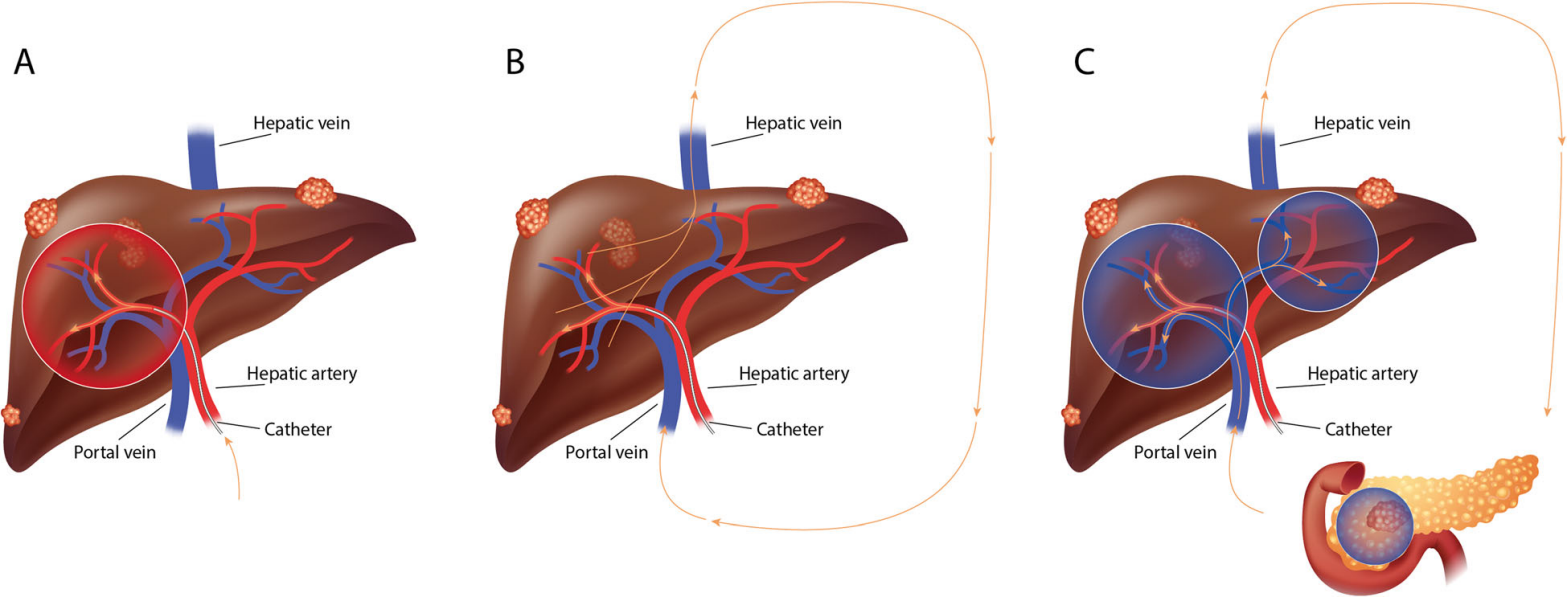
Treatment principle. **a** IA administration of ^177^Lu-DOTATATE selectively in the right or left hepatic artery (right in this figure). **b** Systemic circulation of ^177^Lu-DOTATATE after IA administration. **c** Treatment of whole liver (second-pass) and other organs

The Lutetium Intra-Arterial (LUTIA) study is a multicenter, open-label, phase II, within-patient RCT. The study design differs from the standard parallel-group design, in which patients are allocated by randomization to either the treatment or the control group [16]. Instead, patients will act as their own control, by treating only half of the liver by IA administration of ^177^Lu-DOTATATE. Via the systemic circulation, the contralateral lobe and extrahepatic disease will be treated as if by IV administration (second-pass effect; see Fig. 1). ^177^Lu-DOTATATE activity in tumors in the IA-treated lobe will be compared with the activity in tumors in the contralateral lobe (IV-treated).

**Fig. 2:**
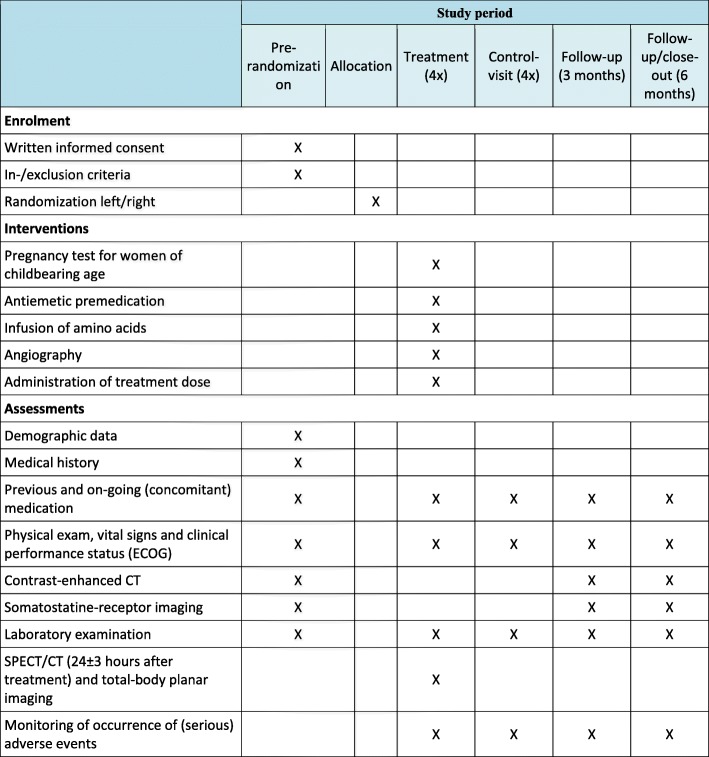
SPIRIT figure showing all phases of the trial, including the interventions and assessment time points

The study will be conducted in accordance with the Declaration of Helsinki and current guidelines of Good Clinical Practice. The current research protocol has been approved by the central Research Ethics Committee of the University Medical Center Utrecht (reference approval number 17-446), and we will not begin recruiting at other centers in the trial until local ethical approval has been obtained. The study protocol has also been approved by the radiation protection committees of the participating centers (i.e., University Medical Center Utrecht, Erasmus Medical Center Rotterdam, Antoni van Leeuwenhoek Amsterdam). A Standard Protocol Items: Recommendations for Interventional Trials (SPIRIT) checklist is provided in Additional file 1. Figure 2 shows the schedule of interventions and assessments [17].

### Subjects

All patients with World Health Organization (WHO) grade I or II NET, originating from gastric, enteric, or pancreatic origin with hepatic metastases and an indication for PRRT are eligible for inclusion. The total hepatic burden should be estimated to involve at least 25% of the liver volume, with a minimum of one lesion of at least 3 cm in size in each liver lobe on cross-sectional imaging. A size of at least 3 cm is required for reliable measurement of activity on single photon emission computed tomography (SPECT)/computed tomography (CT).

### Recruitment

Patients will be recruited in three centers (European Neuroendocrine Tumor Society [ENETS] Centers of Excellence) in The Netherlands. During a multidisciplinary tumor board, patients will be reviewed for eligibility to participate in the study. Study information will be provided by the study physician, nuclear medicine physician, or oncologist/endocrinologist. General inclusion and exclusion criteria for PRRT, as described in the ENETS guidelines, will be applied (Table 1) [1, 18].

**Table 1:** Detailed inclusion and exclusion criteria

Inclusion criteria	Exclusion criteria
Patients must have given written informed consent	Any previous radioembolization, chemoembolization, bland embolization, or external beam radiation therapy to the liver, at any time, or surgery or radiofrequency ablation (or other ablative therapies) within 12 weeks prior to randomization in the study
Male or female aged 18 years or older	Interferons, everolimus (mTOR-inhibitors), or other systemic therapies within 4 weeks prior to randomization in the study
Inoperable histologically proven neuroendocrine tumor with an indication for ^177^Lu-DOTATATE	Use of octreotide or octreotide LAR, if it cannot be interrupted for 24 h or 4 weeks before therapy, respectively, unless tumor uptake on somatostatin receptor imaging is higher than normal liver uptake
Ki-67 index ≤ 20% and a mitotic count of ≤ 20	Unresolved toxicity from previous anti-cancer therapy greater than grade 2
Confirmed presence of somatostatin receptors on target lesions	Serum bilirubin > upper limit of normal (ULN), serum albumin < 3.0 g/dL
Life expectancy of 6 months or longer	Glomerular filtration rate < 50 ml/min
Eastern Cooperative Oncology Group (ECOG) performance score 0–1	Hb < 5.5 mmol/L; leucocytes < 3.0 × 10^9^/L; platelets < 100 × 10^9^/L (at baseline; 75 × 10^9^/L is sufficient for cycles 2–4)
At least one lesion ≥ 3 cm on cross-sectional imaging in both the right and left liver lobes	Uncontrolled congestive heart failure or diabetes mellitus
Presence of excessive liver metastases, defined as > 25% tumor burden	Patients suffering from diseases with an increased chance of liver toxicity
Patients must have clinical or radiological progressive disease	Patients declared incompetent or suffering from psychic disorders making comprehensive judgement impossible
Negative pregnancy test for women of childbearing potential	Previous enrollment in the present study or previous treatment with ^177^Lu-DOTATATE
	Female patients who are not using an acceptable method of contraception, OR are less than 1 year postmenopausal or surgically sterile
	Male patients who are not surgically sterile or do not use an acceptable method of contraception
	Body weight more than 150 kg
	Current spontaneous urinary incontinence
	Severe allergy for intravenous contrast

### Study procedures

#### Screening

**Fig. 3:**
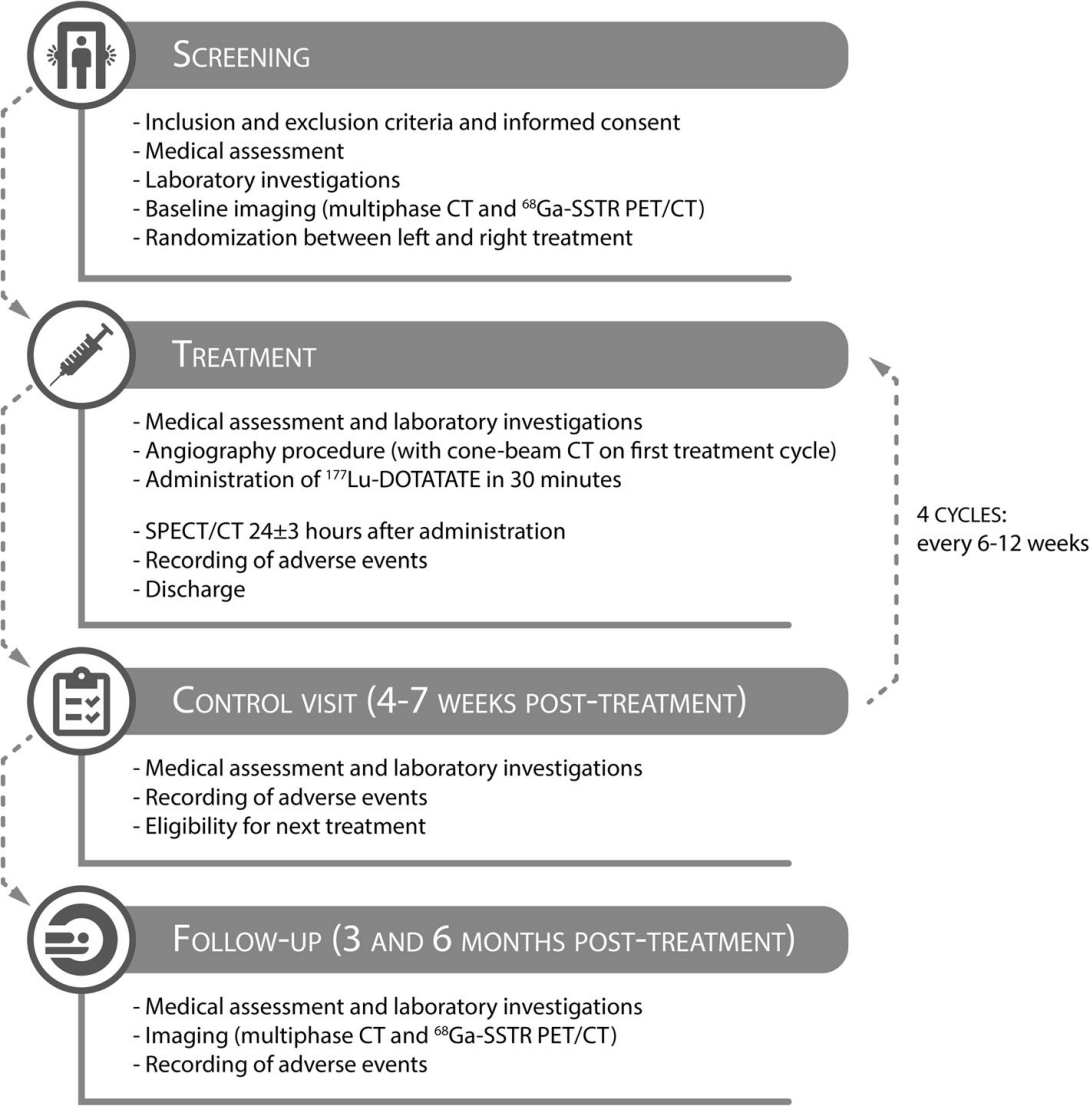
Study procedures in the LUTIA trial

At baseline, demographic data, medical history, medication usage, current complaints, physical examination, and WHO performance status will be recorded (Fig. 3). Furthermore, patients will undergo the following investigations: ^68^Ga-labeled somatostatin analog positron emission tomography/computed tomography (^68^Ga-SSTR PET/CT) to ascertain sufficient somatostatin receptor expression; contrast-enhanced diagnostic CT of the liver to acquire hepatic tumor size and location; laboratory investigations to assess the general condition and presence of toxicity (i.e., liver enzyme and bilirubin levels, coagulation tests, kidney function, and hematologic tests).

#### Treatment

After providing informed consent, patients will be randomized to IA treatment of either the left or the right liver lobe. A complete treatment consists of four cycles of ^177^Lu-DOTATATE every 6 to 12 weeks [18]. On the day of treatment, patients will be hospitalized for one day and one night. Laboratory examination will be performed to ensure no toxicities have occurred and that the patient is fit for treatment and angiography. Prior to or at the start of the angiography procedure, co-infusion of amino acids (1 L 2.5% lysine/arginine during 4 h) is initiated and prophylactic oral anti-emetics (ondansetron 8 mg) are given. During the angiography, the allocated hepatic artery (i.e., left or right) will be catheterized via a femoral or radial approach. A nuclear medicine physician and interventional radiologist will determine the final injection position during angiography. In the first angiographic procedure, a cone-beam computed tomography (CBCT) scan will be performed with a catheter position identical to the injection position. The CBCT confirms the target tumor’s arterial blood supply and demonstrates the arteries’ perfusion territory. Next, 7400 MBq of ^177^Lu-DOTATATE is administered in 30 min, according to the instructions for use of Lutathera [19]. During every cycle, the same injection position is used (i.e., the same randomly allocated liver lobe is IA-treated four times).

All (serious) adverse events will be monitored and logged by medical personnel. A post-treatment ^177^Lu-DOTATATE SPECT/CT scan and total-body planar scintigraphy will be acquired 24 h (± 3 h) post-injection. The acquisition of the SPECT/CT scan after the first cycle is essential for the main study objective, while the SPECT/CT scans after the second, third, and fourth cycles will provide information on the secondary study objectives.

#### Follow-up

A control visit will be scheduled 4 to 7 weeks after each administration. At 3 and 6 months after completion of all four cycles, a control visit is scheduled and a contrast-enhanced diagnostic CT in combination with a ^68^Ga-DOTATOC PET/CT is performed. During all visits, laboratory investigations are performed to assess biochemical and hematological toxicity. Furthermore, patients will undergo physical examination and WHO performance status assessment.

### Objectives

The primary objective is to evaluate whether there is a difference in T/N activity concentration ratio on post-treatment SPECT/CT between the IA- and IV-treated liver lobes. As secondary objectives, activity concentration ratios and absolute mean values of tumor- and healthy liver-absorbed doses in the two lobes will be calculated and compared. Other secondary objectives include the post-treatment tumor response, dose-response relation, and post-treatment hepatic, hematologic, and renal toxicity. Activity concentration in contralateral liver tumors and extrahepatic tumor depositions and renal activity will also be assessed.

### Outcome assessment

To determine the T/N activity ratio on post-treatment SPECT/CT, volumes of interest (VOIs) are drawn based on the pre-treatment contrast-enhanced CT or magnetic resonance imaging (MRI) scan. Only tumors with a minimal diameter of 3 cm are delineated, up to three tumors per liver lobe. For the normal liver tissue VOIs, a representative portion of the normal liver tissue is delineated in each liver lobe, with a minimal diameter of 3 cm. Of all delineated VOIs, the mean number of counts per voxel is calculated.

For the secondary endpoints, a dose-point kernel in commercially available software will be used to calculate the mean absorbed dose, based on the 24-h post-treatment SPECT/CT and mean effective half-life of ^177^Lu-DOTATATE [20, 21]. Comparisons are made between the IA and IV groups by means of a Student *t* test or Mann-Whitney *U* test, where appropriate. Tumor response is assessed on contrast-enhanced CT or MRI using the Southwest Oncology Group (SWOG) solid tumor response criteria, Response Evaluation Criteria In Solid Tumors version 1.1 (RECIST 1.1) and modified Response Evaluation Criteria in Solid Tumors (mRECIST) [22–24]. Response to treatment is assessed on both the patient and liver levels. Moreover, biochemical response will be evaluated using chromogranin A (CgA) levels, measured at baseline and during follow-up. Biochemical, hematological, and clinical toxicity will be graded according to the Common Toxicity Criteria on Adverse Events (CTCAE) version 4.03 [25].

### Sample size assessment

The intended sample size is calculated using a paired samples *t* test on the difference in T/N uptake ratio between the IA-treated and contralateral lobes, which is equivalent to a one-sample *t* test on the within-patient difference scores. Assuming a moderate to large effect (i.e., $\text{d}_{z}={\mu}_{diff}/{\sigma}_{diff}= 0.65$), a one-sided α of 0.025, and using a sequential testing procedure with an O’Brien-Fleming type error spending function, a power of approximately 0.9 is obtained with futility boundary values equal to T_1_ = − 0.1045056 and T_2_ = 2.045732 for an interim analysis at n_1_ = 10 and final analysis at n_2_ = 26. Interim analysis will therefore be performed after ten patients have been treated, and final analysis will be performed after 26 patients have been treated.

### Randomization

For randomizing patients between left or right IA treatment, an online randomization tool is used to allow for multicenter access. The randomization algorithm is a computer-generated permuted block sequence with different block sizes. Due to the relatively small study sample (*n* = 26), only small blocks of size = 1 and size = 2 are used. No stratification is used.

### Statistical analysis

Continuous variables, such as the primary outcome (i.e., T/N ratio), will be described by means and standard deviations. The mean T/N ratios will be compared between the IA- and IV-treated lobes by means of a paired *t* test. Categorical variables, such as the response after treatment, will be compared by means of a chi-square or Fisher’s exact test, depending on group size.

All statistical tests will be performed two-sided. A *p* value of < 0.05 will be considered statistically significant.

## Discussion

Currently, many patients presenting with metastasized NETs experience significantly improved PFS and OS after treatment with ^177^Lu-DOTATATE. However, patients with diffuse and bulky liver metastases still have a worse prognosis and a lower disease control rate after treatment with PRRT, which leaves room for improvement of PRRT. The post hoc analysis of the NETTER-1 trial reported that the presence of bulky disease significantly limits median PFS after treatment with ^177^Lu-DOTATATE to 28 months, while the median PFS was not reached in 5 years of follow-up in patients with no bulky disease [14]. Of those patients with bulky disease, 70% had bulky liver disease. Earlier, Kwekkeboom et al. reported that a high hepatic tumor burden significantly reduced the median disease-specific survival from more than 48 months to only 25 months [13]. In line with their results, Ezziddin et al. reported a median OS of 43 months in patients with a hepatic tumor burden of more than 25%, while median OS was not reached (> 70 months) in patients with a hepatic tumor burden < 25% [12]. To increase the concentration of ^177^Lu-DOTATATE in intrahepatic tumors, IA administration may be an effective and relatively easy improvement to boost patient outcome. In the current study, the beneficial effect of IA ^177^Lu-DOTATATE will be studied in a controlled design.

To date, no prospective study on IA administration has been performed. A small number of studies, using different radiopharmaceuticals, have shown a beneficial effect [15]. The first study, performed by McStay et al*.*, showed no additional toxicity when administering yttrium-90 (^90^Y)-DOTA-lanreotide IA [26]. In three publications by Limouris and colleagues, promising results were reported with response rates of ~ 50% [27–29]. Kratochwil et al. found a partial response in 8/15 patients and a complete response in 1/15 patients after IA treatment with a combination of ^90^Y- and ^177^Lu-DOTATOC [30]. The same authors previously reported significantly enhanced tumor uptake of the diagnostic radiotracer ^68^Ga-DOTATOC after IA administration [31]. In a non-randomized crossover-like study, they performed a ^68^Ga-DOTATOC PET/CT after IV administration and, subsequently, a ^68^Ga-DOTATOC PET/CT after IA administration. Standardized uptake values in NETs were approximately 3.75 times higher after IA administration. Comparable enhancement of activity concentration in liver metastases was also shown by Pool et al., in a similar crossover-like study in three patients treated with indium-111-DTPA-octreotide, both IV and IA [32]. Up to a 2.4-fold higher concentration in tumors was quantified. In the available literature, no additional side effects were observed.

To our knowledge, the LUTIA study is the first RCT to investigate the effect of IA administration of ^177^Lu-DOTATATE in patients with NETs. Previously performed studies lacked comparison with IV administration. In the LUTIA study, a direct comparison can be made between IV and IA administration within the same patient. Consequently, between-patient differences have little to no effect on the difference in T/N activity ratios. This is a great advantage of this study design with within-patient randomization, since NETs are known to be very heterogeneous. The heterogeneity of tumor characteristics (such as tumor subtype, growth rate, SSTR-2 expression, functional state, and imaging characteristics) causes difficulty when performing comparative research on NETs [33]. Within-patient comparison automatically corrects for any differences in tumor characteristics between patients. Furthermore, the paired design increases power in statistical analysis, and fewer patients need to be treated to notice an effect.

Multiple alternatives can be adopted in boosting treatment of NET liver metastases. For example, trans-arterial chemo or bland embolization (TACE/TAE) and radioembolization (TARE) are applied in loco-regional management of liver metastases. As liver metastases have a considerable effect on quality of life and survival, improving treatment of NET liver metastases has rightfully gained attention [34, 35]. Due to the multiple treatment strategies available, a patient-tailored approach is desirable. An increasingly used liver-directed therapy is TARE with ^90^Y-loaded glass or resin microspheres. Objective response rates of approximately 50% have been reported, and disease control is achieved in approximately 80% of patients with NETs [36–38]. The difference between TARE, TACE, and TAE is controversial, since similar PFS has been obtained. However, side effects are probably more likely to occur after TACE/TAE [1, 34, 39, 40]. Even though the most recent ENETS guidelines suggest that loco-regional therapies can be applied in the absence of extrahepatic disease, more and more studies suggest that there is room for radioembolization even though extrahepatic disease is present [1, 34, 37, 41]. When extrahepatic disease is present, a combined or sequential application of TARE with PRRT might be of added value in the future [42–44].

There are some limitations to the current study. First, the number of subjects who will be included is rather small (i.e., 26). As a result, a small effect can possibly be missed. However, to justify the additional angiographic procedure and obtain a clinically relevant effect, the increase in T/N ratio should be rather large. This increase will be noticeable with a study sample of 26 patients. Second, the second-pass effect used as a proxy for IV administration in this study might not be exactly the same as for regular IV administration, because a larger fraction of ^177^Lu-DOTATATE may be absorbed by IA-treated tumors. However, an excess of ^177^Lu-DOTATATE is administered (the same dose as in regular treatment) considering the relatively high urinary excretion of activity. This effect is therefore estimated to be negligible.

This study will provide insights in the IA administration of ^177^Lu-DOTATATE in a prospective and controlled design. The LUTIA study will investigate the potential benefit of IA administration of ^177^Lu-DOTATATE instead of conventional IV PRRT in patients with NET liver metastases. Increased activity concentration in liver metastases may lead to better response and survival. Positive study results will lead to a large RCT, investigating the long-term outcome of IA PRRT. Implementation of IA PRRT will need to be part of a personalized-medicine approach for the patient, so that only patients benefitting from IA PRRT will be treated.

## Trial status

Patient recruitment was ongoing at the time of submission. The protocol version is 3.0; recruitment started on 27 June 2018; estimated completion of recruitment will be achieved in the third quarter of 2020.

## Supplementary information

**Additional file 1.** Standard Protocol Items: Recommendations for Interventional Trials (SPIRIT) 2013 checklist: recommended items to address in a clinical trial protocol and related documents.

## Data Availability

Not applicable.

## Abbreviations

^177^Lu-DOTATATE: Lutetium-177-DOTA-octreotate
^90^Y: Yttrium-90
CBCT: Cone-beam computed tomography
CgA: Chromogranin A
CTCAE: Common Toxicity Criteria on Adverse Events
ENETS: European Neuroendocrine Tumor Society
IA: Intra-arterial
IV: Intravenous
LUTIA: Lutetium Intra-Arterial
mRECIST: Modified Response Evaluation Criteria In Solid Tumors
NET: Neuroendocrine tumor
OS: Overall survival
PET/CT: Positron emission tomography/computed tomography
PFS: Progression-free survival
PRRT: Peptide receptor radionuclide therapy
RCT: Randomized controlled trial
RECIST 1.1: Response Evaluation Criteria In Solid Tumors version 1.1
SPECT/CT: Single photon emission computed tomography/computed tomography
SSTR-2: Somatostatin receptor subtype 2
SWOG: Southwest Oncology Group
T/N: Tumor-to-non-tumor
TACE: Trans-arterial chemoembolization
TAE: Trans-arterial embolization
TARE: Trans-arterial radioembolization
VOI: Volume of interest
WHO: World Health Organization
